# Treatment Outcomes of Extensively Drug-Resistant Tuberculosis in Pakistan: A Countrywide Retrospective Record Review

**DOI:** 10.3389/fphar.2021.640555

**Published:** 2021-03-31

**Authors:** Muhammad Abubakar, Nafees Ahmad, Abdul Ghafoor, Abdullah Latif, Izaz Ahmad, Muhammad Atif, Fahad Saleem, Shereen Khan, Amjad Khan, Amer Hayat Khan

**Affiliations:** ^1^ Department of Pharmacy Practice, Faculty of Pharmacy and Health Sciences, University of Balochistan, Quetta, Pakistan; ^2^ MDR-TB Specialist, National TB Control Program, Islamabad, Pakistan; ^3^ Data Manager, National AIDS, TB and Malaria Control Program, Islamabad, Pakistan; ^4^ Department of Biology, Syed Babar Ali School of Science and Engineering, Lahore University of Management Sciences, Lahore, Pakistan; ^5^ Department of Pharmacy Practice, Faculty of Pharmacy, The Islamia University of Bahawalpur, Bahawalpur, Pakistan; ^6^ Bolan Medical College, Quetta, Pakistan; ^7^ Department of Pharmacy, Quaid-i-Azam University, Islamabad, Pakistan; ^8^ Discipline of Clinical Pharmacy, School of Pharmaceutical Sciences, University Sains Malaysia, Penang, Malaysia

**Keywords:** death, high-dose isoniazid, sputum culture conversion, treatment outcomes, XDR-TB

## Abstract

**Background:** The current study is conducted with the aim to the fill the gap of information regarding treatment outcomes and variables associated with unsuccessful outcome among XDR-TB patients from Pakistan.

**Methods:** A total of 404 culture confirmed XDR-TB patients who received treatment between 1^st^ May 2010 and June 30, 2017 at 27 treatment centers all over Pakistan were retrospectively followed until their treatment outcomes were reported. A *p*-value <0.05 reflected a statistical significant association.

**Results:** The patients had a mean age 32.9 ± 14.1 years. The overall treatment success rate was 40.6% (95% confidence interval [CI]:35.80–45.60%). A total of 155 (38.4%) patients were declared cured, 9 (2.2%) completed treatment, 149 (36.9%) died, 60 (14.9%) failed treatment and 31 (7.7%) were lost to follow up (LTFU). The results of the multivariate binary logistic regression analysis revealed that the patients’ age of >60 years (OR = 4.69, 95%CI:1.57–15.57) and receiving high dose isoniazid (OR = 2.36, 95%CI:1.14–4.85) had statistically significant positive association with death, whereas baseline body weight >40 kg (OR = 0.43, 95%CI:0.25–0.73) and sputum culture conversion in the initial two months of treatment (OR = 0.33, 95%CI:0.19–0.58) had statistically significant negative association with death. Moreover, male gender had statistically significant positive association (OR = 1.92, 95%CI:1.04–3.54) with LTFU.

**Conclusion:** The treatment success rate (40.6%) of XDR-TB patients in Pakistan was poor. Providing special attention and enhanced clinical management to patients with identified risk factors for death and LTFU in the current cohort may improve the treatment outcomes.

## Introduction

Extensively drug-resistant *tuberculosis* (XDR-TB) is defined as “TB caused by a strain of *Mycobacterium tuberculosis* (MTB) concurrently resistant to isoniazid, rifampicin, a fluoroquinolone (FQ) and a second-line injectable anti-TB drug (SLI) i.e., amikacin/kanamycin/capreomycin” (World Health Organization, 2014). It was first reported in 2005, and to date, 123 countries have notified at least one patient suffering from XDR-TB (World Health Organization, 2020). In 2019, a total of 12,350 XDR-TB patients were notified worldwide, and on average 6.2% multidrug resistant TB (MDR-TB) patients have XDR-TB (World Health Organization, 2020). The concurrent resistance to the four most effective first and second anti-TB drugs i.e., rifampicin, isoniazid, an SLI and a FQ, leaves XDR-TB patients to be treated for prolonged periods with a large number of less effective and more toxic drugs, consequently resulting in higher morbidity and mortality. Globally, the treatment success rates for drug susceptible TB, MDR-TB and XDR-TB are 85% (2017 cohort), 56% (2017 cohort) and 39% (2016 cohort), respectively (World Health Organization, 2018; World Health Organization, 2020). The previously reported treatment success rate of various individual cohorts of XDR-TB patients (n = 12–195) ranges from 4 to 65% (Abbate et al., 2012; Alene et al., 2017; He et al., 2017; Prajapati et al., 2017; Gallo et al., 2018; Yuengling et al., 2018; Frank et al., 2019; Makhmudova et al., 2019; Te Riele et al., 2019).

Unfortunately, with an estimated incidence of 28,000 MDR-TB patients, Pakistan is a DR-TB 5^th^ high burden country in the world (World Health Organization, 2020). Pakistan initiated the programmatic management of drug resistant TB (PMDT) in 2010 (Ahmad et al., 2015), and at present there are 33 functional PMDT units all over the country. Since the inception of PMDT in 2010 and until June 30, 2019, a total of 560 XDR-TB patients have been enrolled on treatment at 30 PMDT units throughout country. Under operational conditions, evaluating a cohort of patients for treatment outcomes is a conventional, widely employed and effective method for examining the effectiveness of a program and treatment regimen (Kurbatova et al., 2012). The reported treatment success rate of a number of cohorts of MDR-TB patients treated at various PMDT units in Pakistan ranges from 40.5 to 76.9% (Ahmad et al., 2015; Javaid et al., 2017; Javaid et al., 2018; Khan et al., 2019). However, there was a complete lack of information about treatment outcomes and factors associated unsuccessful treatment outcomes among XDR-TB patients from Pakistan. Therefore, the current research was conducted to assess the treatment outcomes and factors associated with unsuccessful outcomes among XDR-TB patients in Pakistan.

## Materials and Methods

### Study Design, Settings and Population

The current study was a retrospective record review of all culture confirmed XDR-TB patients who received treatment between 1^st^ May 2010 and June 30, 2017 at 27 PMDT units across the country (Supplementary Table S1).

### Diagnosis and Treatment of XDR-TB Patients

In compliance with National TB Control Program (NTP) guidelines, all presumed DR-TB patients sent to the PMDT units were evaluated for the presence of MTB, resistance to rifampicin and isoniazid by examining two sputum samples via direct sputum smear microscopy, Xpert MTB/Rif (Cepheid, Sunnyvale, CA, United States) and line probe assay, respectively. After the diagnosis of rifampicin-resistance TB through these tests, patients were enrolled on empirical MDR-TB treatment regimen and their diagnostic samples were sent to national or provincial reference laboratories for drug susceptibility testing (DST) against first-line anti-TB drugs (FLD) and second-line anti-TB drugs (SLD). At these reference laboratories, Agar proportion method on enriched Middlebrook 7H10 medium (BBL; Beckton Dickinson, Sparks, MD, United States) at the following concentrations was used for conducting DST of FLD and SLD: isoniazid (0.2 μg/mL), rifampicin (1 μg/mL), ethambutol (5 μg/mL), streptomycin (2 μg/mL), amikacin, (4 μg/mL), kanamycin (5 μg/mL), capreomycin (4 μg/mL), ofloxacin (2 μg/mL), levofloxacin (1 μg/mL) and ethionamide (5 μg/mL). Complying with manufacturer’s instructions, DST for pyrazinamide was conducted by using BACTEC Mycobacterial Growth Indicator Tube (MGIT, BD, Sparks, MD, United States) at a concentration of 100 μg/mL (Javaid et al., 2018). When the DST results became available, XDR-TB patients were switched to an individualized treatment regimen devised on the basis of DST results and guidelines recommendations (National TB Control Program, 2014; World Health Organization, 2014). They were treated with an individualized longer treatment regimen mainly comprised of an SLI, preferably the one to which the strain is sensitive, a high generation FQ (levofloxacin/moxifloxacin), all available likely effective Group-4 SLD (etionamide, cycloserine and para-amino salicylic acid), ethambutol if the strain was sensitive, pyrazinamide and two or more of the Group-5 drugs (bedaquiline, delamanid, linezolid, clofazimine, amoxicillin/clavulanate, imipenem/cilastatin + clavulanate, meropenem + clavulanate, high-dose isoniazid, clarithromycin, thioacetazone). The total duration of treatment was a minimum of 20 months with at least 18 months after sputum culture conversion (SCC) defined as “two consecutive negative cultures taken at the gap of at least 30 days following an initial positive culture” (National TB Control Program, 2014; World Health Organization, 2014). SLIs were used for a minimum of 8–12 months. All the included XDR-TB patients were treated as outpatients throughout the treatment. Their adherence with the treatment regimen was monitored by trained treatment supporters, evaluated by the doctors on monthly visits and ensured by a home DOTS (directly observed treatment, short-course) linkage facilitator by visiting their homes, and connecting the patients, nearby healthcare facilities, the district TB officers and the PMDT units. All the patients received free of cost treatment. Moreover, monthly food rations and transportation charges were given to all patients and their treatment supporters.

### Data Collection

Every month, the data of DR-TB patients treated at PMDT centers is shared with NTP through an electronic nominal recording and reporting system (ENRS). ENRS is the electronic version of the main four registers in the TB recording reporting system, i.e., basic management unit TB register, second-line TB treatment register, laboratory register for smear microscopy and Xpert MTB/Rif and laboratory register for culture, Xpert MTB/RIF and DST. In ENRS, data are entered on nominal bases at PMDT units using Excel and processed to produce routine reports to the NTP and to calculate indicators. We used a purpose developed data collection form (appendix A) to extract the sociodemographic, microbiological and clinical data of XDR-TB patients from the ENRS shared with NTP. WHO guidelines were used to categorize patients’ end treatment outcomes (Supplementary Table S2) (World Health Organization, 2014). The treatment outcomes of death, treatment failure and lost to follow-up (LTFU) were grouped together as unsuccessful outcomes, whereas, cure and treatment completed were grouped together as successful outcomes (World Health Organization, 2014).

### Statistical Analysis

Statistical Package for Social Science (version 23, IBM Corp., Armonk, N.Y., United States) was used for analyzing data. Univariate analysis was used to asses association between patients’ sociodemographic, microbiological and clinical variables and unsuccessful treatment outcomes i.e., death, LTFU and treatment failure. The entry of the independent variables in univariate analysis was based on the previously published studies, their possible relationship with the treatment outcomes and recommendations from the clinical team and supervisors of the current study. In order to find final factors associated with death, LTFU and treatment failure, multivariate binary logistic regression (MVBLR) analysis was conducted. Independent variables with a *p*-value <0.2 in univariate analysis were carried forward to MVBLR analysis. Independent variables with a high correlation (tolerance value < 0.1 and/or variance inflation factor 10) were not included in MVBLR analysis. Statistical significance was taken at a *p*-value of <0.05.

### Ethical Approval

Ethical approval was taken from Research and Ethics Committee of the Faculty of Pharmacy and Health Sciences, University of Balochistan Quetta and NTP Islamabad (Ref: DRF04/12/19).

## Results

**FIGURE 1:**
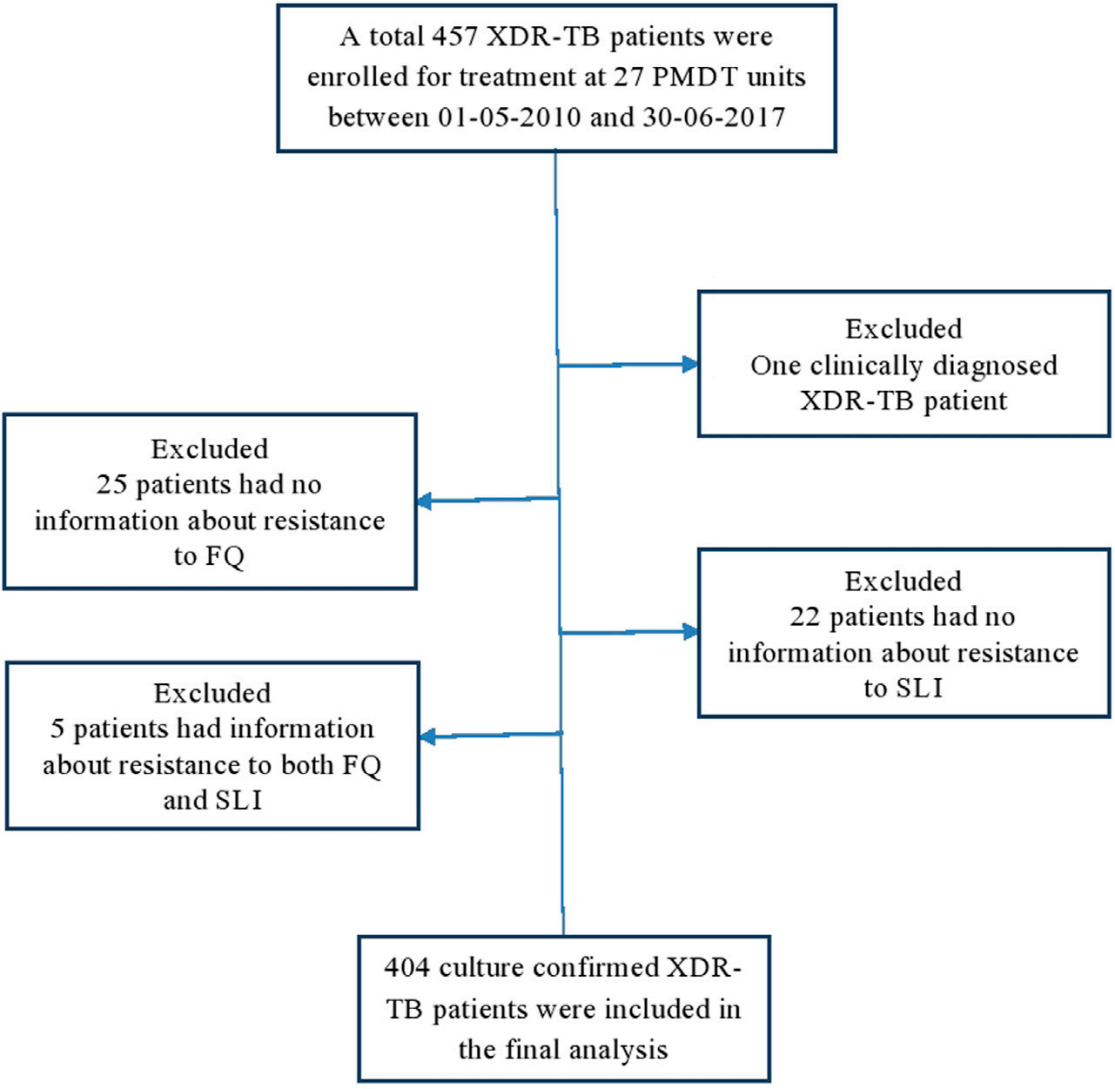
Enrolment, inclusion and exclusion of study participants. DST, drug susceptibility testing; FQ fluoroquinolones; SLI, second-line injectables; XDR-TB, extensively drug resistant tuberculosis

Between 01-05-2010 and 30-06-2017, a total of 457 XDR-TB patients received treatment at 27 PMDT units in Pakistan (Supplementary Table S1). Out of these 457 patients, the complete drug resistance pattern information was available for 404 patients and they were included in the current study (Figure 1). The mean age of patients was 32.9 ± 14.1 years. The results of cross-tabulation between patients’ sociodemographic, clinical and microbiological characteristics and treatment outcomes are given in Table 1. Patients were resistant to a median of 7 drugs (interquartile range [IQR] = 6–8). A total of 182 (45.5%) patients were concurrently resistant to all the three SLIs and 166 (41.1%) to all the five FLDs (Table 2). Out of 397 pulmonary XDR-TB patients, SCC was achieved by 244 patients (61.5%). Median time to SCC was 3 months (IQR = 2–5 months). Among 244 patients who achieved SCC, 110 (45%) were culture converted by second month of treatment, 170 (69.7%) by 4th and 206 (84.4%) by 6th month of treatment.

**TABLE 1:** Patients’ socio-demographic, clinical and microbiological characteristics and treatment outcomes.

Variable	No. (%)	Treatment outcomes		*p*-value
		Successful	Unsuccessful	
Gender				0.525
Female	182 (45.0)	77 (42.3)	105 (57.7)	
Male	222 (55.0)	87 (39.2)	135 (60.8)	
Age (years)				0.045
≤20	87 (21.5)	45 (51.7)	42 (48.3)	
21–40	207 (51.2)	81 (39.1)	126 (60.9)	
41–60	91 (22.5)	34 (34.7)	57 (62.6)	
>60	19 (4.7)	4 (21.1)	15 (78.9)	
Baseline body weight (Kg)				0.012
<40	140 (34.7)	45 (32.1)	95 (67.9)	
≥40	260 (65.3)	119 (45.1)	145 (54.9)	
Smoking				0.058
No	381 (94.3)	159 (41.7)	222 (58.3)	
Yes	23 (5.7)	5 (21.7)	18 (78.3)	
Comorbidity				0.281
No	342 (84.7)	135 (39.5)	207 (60.5)	
Yes	62 (15.3)	29 (46.8)	33 (53.2)	
Type of comorbidities				
Diabetes mellitus	30			
Psychiatric disorders	9			
Liver diseases	9			
HIV	1			
Others	13			
History of TB treatment				0.006
No	26 (6.4)	15 (57.7)	11 (42.3)	
Yes	378 (93.6)	149 (39.4)	229 (60.6)	
History of SLD use				0.516
No	251 (62.1)	105 (48.1)	146 (58.2)	
Yes	153 (37.9)	59 (38.6)	94 (61.4)	
History of MDR-TB treatment				0.346
No	260 (64.4)	110 (42.3)	150 (57.7)	
Yes	144 (35.6)	54 (37.5)	90 (62.5)	
Site of TB				0.125^a^
Pulmonary	397 (98.3)	159 (40.1)	238 (59.5)	
Extra-pulmonary	7 (1.7)	5 (71.4)	2 (28.6)	
Baseline smear grading				0.145
Negative	73 (18.1)	36 (49.3)	37 (50.7)	
Scanty,^b^ +1^c^	122 (30.2)	54 (44.3)	68 (55.7)	
+2^d^, +3^e^	199 (49.4)	70 (35.2)	129 (64.8)	
Information not available	10 (2.3)	4 (40.0)	6 (60.0)	

FLD, first-line anti-TB drugs; Kg, kilogram; MDR-TB, multidrug resistant TB; SLD, first-line anti-TB drugs.

^a^ Fisher exact test.

^b^ Scanty = 1–9 AFB (Acid fast bacilli)/100 HPF (High power field).

^c^ +1 = 10–99 AFB/100 HPF).

^d^ +2 = 1–9 AFB/HPF.

^e^ +3 > 9 AFB/HPF.

**TABLE 2:** Drug resistance pattern of study participants.

Variable	No. (%)
No resistant drugs at baseline visit	
Four drugs	15 (3.7)
Five drugs	40 (9.9)
Six drugs	55 (13.6)
Seven drugs	96 (23.8)
Eight drugs	99 (24.5)
Nine drugs	83 (20.5)
Ten drugs	15 (3.7)
Eleven drugs	1 (0.2)
Resistance pattern to FLDs	
Isoniazid + rifampicin	33 (8.2)
Isoniazid + rifampicin + ethambutol	17 (4.2)
Isoniazid + rifampicin + pyrazinamide	38 (9.4)
Isoniazid + rifampicin + streptomycin	19 (4.7)
Isoniazid + rifampicin + ethambutol + pyrazinamide	70 (17.3)
Isoniazid + rifampicin + ethambutol + streptomycin	46 (11.4)
Isoniazid + rifampicin + pyrazinamide + streptomycin	15 (3.7)
All five FLD	166 (41.1)
Resistance to SLDs	
Resistance to SLI + fluoroquinolone	355 (87.9)
Resistance to SLI + fluoroquinolone + ethionamide	49 (12.1)
Resistance to amikacin	306 (75.7)
Resistance to kanamycin	305 (75.5)
Resistance to capreomycin	253 (62.6)
Concurrent resistance to all three SLIs	182 (45.5)

FLD, first-line anti-TB drugs; SLD, second-line anti-TB drugs; SLI, second-line injectables.

### Treatment Outcomes and Factors Associated With Unsuccessful Outcomes

In the current study, a total of 40.6% (95% confidence interval [CI]:35.80–45.60%) were treated successfully. Out of 404 patients included in the final analysis, 155 (38.4%) were declared cured, 9 (2.2%) completed treatment, 149 (36.9%) died, 60 (14.9%) treatment failures and 31 (7.7%) as LTFU. The median time to death was 5 months (range 1–30 months). Out of 149 patients who died, 120 died during the first 12 months of treatment. Out of 31 LTFU patients, 29 were LTFU prior to completing the intensive phase of treatment and 24 were culture positive at the time of LTFU.

Upon MVBLR analysis, The results of the multivariate binary logistic regression analysis revealed that the patients’ age of >60 years (OR = 4.69, 95%CI:1.57–15.57, *p*-value = 0.006) and receiving high dose isoniazid (OR = 2.36, 95%CI:1.14–4.85, *p*-value = 0.019) had statistically significant positive association with death, whereas baseline body weight >40 kg (kg) (OR = 0.43, 95%CI:0.25–0.73, *p*-value = 0.002) and sputum culture conversion in the initial two months of treatment (OR = 0.33, 95%CI:0.19–0.58, *p*-value<0.001) had statistically significant negative association with death (Table 3). Moreover, male gender had statistically significant positive association (OR = 1.92, 95%CI:1.04–3.54, *p*-value = 0.037) with LTFU (Table 4). However, the results of MVBLR analysis did not reveal any statistical significant association between independent variables and treatment failure (Table 5).

**TABLE 3:** Factors associate with death.

Variable	Died n (%)	Univariate analysis OR (95%CI)	*p*-value	Multivariate analysis OR (95%CI)	*p*-value
Gender					
Female	78 (42.9)	Referent		Referent	
Male	71 (32)	0.62 (0.41–0.94)	0.025	0.84 (0.51–1.39)	0.510
Age (years)					
≥20	29 (33.3)	Referent		Referent	
21–40	75 (36.2)	1.13 (0.67–1.92)	0.635	1.35 (0.75–2.41)	0.311
41–60	32 (35.2)	1.08 (0.58–2.01)	0.797	1.68 (0.82–3.42)	0.149
>60	13 (68.4)	4.33 (1.49–12.57)	0.007	4.96 (1.57–15.57)	**0.006**
Baseline body weight (kg)					
<40	67 (47.9)	Referent		Referent	
≥40	82 (31.1)	0.49 (0.32–0.74)	0.001	0.43 (0.25–0.73)	**0.002**
Resistance to all five FLD					
No	97 (40.8)	Referent		Referent	
Yes	52 (31.3)	0.66 (0.43–1.00)	0.054	0.68 (0.42–1.11)	0.128
Baseline sputum smear grading					
Negative	22 (30.1)	Referent		Referent	
Scanty+1	41 (33.6)	1.17 (0.62–2.19)	0.616	0.81 (0.40–1.62)	0.544
+2 + 3	84 (42.2)	1.69 (0.95–3.00)	0.072	1.43 (0.75–2.73)	0.277
Not available	2 (20)	0.58 (0.11–2.95)	0.511	0.47 (0.09–2.51)	0.382
SCC at two months of treatment					
No	127 (43.5)	Referent		Referent	
Yes	22 (19.6)	0.31 (0.18–0.53)	<0.001	0.33 (0.19–0.58)	**<0.001**
Receiving clofazimine					
No	62 (33.2)	Referent		Referent	
Yes	87 (40.1)	1.34 (0.89–2.02)	0.150	1.35 (0.83–2.20)	0.222
Receiving high dose isoniazid					
No	129 (35.5)	Referent		Referent	
Yes	20 (48.8)	1.72 (0.90–3.30)	0.099	2.36 (1.14–4.85)	**0.019**

Note: Only those variables are presented in the table which in univariate analysis had a *p*-value <0.2 were entered in multivariate analysis. This model fit was based on non-significant Hosmer lemeshow (*p*-value = 0.268) and overall percentage 66.6% from classification table.

CI, confidence interval; kg, kilogram; OR, odds ratio; SCC, sputum culture conversion; scanty = 1–9 AFB (Acid fast bacilli)/100 HPF (High power field); **+1 = 10–99 AFB/100 HPF); ***+2 = 1–9 AFB/HPF; ****+3 > 9 AFB/HPF.

**TABLE 4:** Factors associated with lost to follow-up.

Variable	LTFU n (%)	Univariate analysis OR (95%CI)	*p*-value	Multivariate analysis OR (95%CI)	*p*-value
Gender					
Female	9 (4.9)	Referent		Referent	
Male	22 (9.9)	2.11 (0.94–4.71)	0.067	1.92 (1.04–3.54)	**0.037**
Age (years)					
≥20	4 (4.6)	Referent		Referent	
21–40	20 (9.7)	2.21 (0.73–6.69)	0.157	1.40 (0.63–3.12)	0.402
41–60	5 (5.5)	1.20 (0.31–4.67)	0.785	1.98 (0.42–4.83)	0.125
>60	2 (10.5)	2.44 (0.41–14.41)	0.325	Non-computable	-
Baseline sputum smear grading					
Negative	4 (5.5)	Referent		Referent	
Scanty^a^, +1^b^	11 (9)	1.70 (0.52–5.58)	0.374	1.00 (0.41–2.42)	0.988
+2^c^, +3^d^	14 (7)	1.30 (0.41–4.10)	0.648	1.24 (0.56–2.71)	0.591
NA	2 (20)	4.31 (0.67–27.38)	0.121	1.30 (0.23–7.29)	0.762
SCC at two months of treatment					
No	29 (9.9)	Referent		Referent	
Yes	2 (1.8)	0.16 (0.03–0.70)	0.015	1.24 (0.63–2.33)	0.497

Note: Only those variables are presented in the table which in univariate analysis had a *p*-value <0.2 were entered in multivariate analysis. This model fit was based on non-significant Hosmer lemeshow (*p*-value = 0.701) and overall percentage of 85.1% from classification table.

CI, confidence interval; kg, kilogram; LTFU, lost to follow up; OR, odds ratio; SCC, sputum culture conversion

^a^ Scanty = 1–9 AFB (Acid fast bacilli)/100 HPF (High power field);

^b^ +1 = 10–99 AFB/100 HPF);

^c^ +2 = 1–9 AFB/HPF;

^d^ +3 > 9 AFB/HPF.

**TABLE 5:** Factors associated with treatment failure.

Variable	Failed n (%)	Univariate analysis OR (95%CI)	*p*-value	Multivariate analysis OR (95%CI)	*p*-value
Gender					
Female	18 (9.9)	Referent		Referent	
Male	42 (18.9)	2.12 (1.17–3.84)	0.012	1.73 (0.85–3.52)	0.126
Age (years)					
≥20	9 (10.3)	Referent		Referent	
21–40	31 (15)	1.52 (0.69–3.35)	0.293	1.43 (0.62–3.28)	0.395
41–60	20 (22)	2.44 (1.04–5.711)	0.040	1.80 (0.71–4.57)	0.216
>60	0 (0)	Non-computable	-	Non-computable	-
Baseline body weight (kg)					
<40	14 (10)	Referent		Referent	
≥40	46 (17.4)	1.89 (1.00–3.59)	0.049	1.27 (0.60–2.71)	0.520
Smoking					
No	54 (14.2)	Referent		Referent	
Yes	6 (26.1)	2.13 (0.80–5.66)	0.127	1.22 (0.39–3.77)	0.729
Resistance to pyrazinamide					
No	12 (10.4)	Referent		Referent	
Yes	48 (16.6)	1.71 (0.87–3.35)	0.119	1.67 (0.73–3.82)	0.223
Resistance to ethionamide					
No	56 (15.8)	Referent		Referent	
Yes	4 (8.2)	0.47 (0.16–1.37)	0.169	0.40 (0.12–1.24)	0.115
Receiving clarithromycin					
No	19 (11)	Referent		Referent	
Yes	41 (17.7)	1.72 (0.96–3.10)	0.066	1.89 (0.96–3.68)	0.062
Receiving bedaquiline					
No	57 (15.7)	Referent		Referent	
Yes	3 (7.3)	0.42 (0.12–1.42)	0.164	0.56 (0.14–2.25)	0.418
Receiving clofazimine					
No	34 (18.2)	Referent		Referent	
Yes	26 (12)	0.61 (0.35–1.06)	0.082	1.01 (0.46–2.23)	0.970
Receiving linezolid					
No	24 (19.7)	Referent		Referent	
Yes	36 (12.8)	0.59 (0.33–1.05)	0.075	0.67 (0.33–1.38)	0.283
Receiving moxifloxacin					
No	26 (19.3)	Referent		Referent	
Yes	34 (12.6)	0.60 (0.34–1.06)	0.080	0.69 (0.32–1.45)	0.327
Number of resistant drugs					
4–6	10 (9.1)	Referent		Referent	
7–8	36 (18.5)	2.26 (1.07–4.76)	0.031	1.82 (0.77–4.32)	0.172
>8	14 (14.1)	1.64 (0.69–3.89)	0.256	1.37 (0.47–3.93)	0.558

Note: Only those variables are presented in the table which in univariate analysis had a *p*-value <0.2 were entered in multivariate analysis. This model fit was based on non-significant Hosmer lemeshow (*p*-value = 0.471) and overall percentage of 84.9% from classification table.

CI, confidence interval; kg, kilogram; OR, odds ratio.

## Discussion

To the best of our knowledge, this is the first study which evaluated the treatment outcomes and factors associated with unsuccessful outcomes among XDR-TB patients enrolled for treatment at 27 PMDT units all over Pakistan. As the previously published individual cohorts from different countries have included a relatively small number of XDR-TB patients ranging from 12 to 195 (Kwon et al., 2008; Kliiman & Altraja, 2009; Pietersen et al., 2015; Yuengling et al., 2018; Makhmudova et al., 2019; Yunusbaeva et al., 2019), the large sample size of 404 XDR-TB patients from all over the country is the major strength of this study. In the present cohort, the treatment success rate (40.6%) was comparable with the global treatment success rate (39%) among XDR-TB patients (2016 cohort) (World Health Organization, 2018). However, it was better than the rates reported from Tajikistan (5.6%) (Makhmudova et al., 2019), Russia (12%) (Yunusbaeva et al., 2019), South Africa (4 and 31.4%) (Yuengling et al., 2018; Te Riele et al., 2019), China (14.6 and 30%) (Alene et al., 2017; He et al., 2017), India (25.9%) (Prajapati et al., 2017) and Georgia (33%) (Frank et al., 2019), and lower than the ones reported from Brazil (48.4%) (Gallo et al., 2018) and *Argentina* (65%) (Abbate et al., 2012).

In the current study, a total of 149 (36.9%) patients died. The mortality rate in our cohort was comparatively higher than the frequency of deaths observed among XDR-TB patients in Georgia (15.09%) (Frank et al., 2019), Estonia (17.54%) (Kliiman & Altraja, 2009) and Russia (23.61%) (Yunusbaeva et al., 2019), and lower than that observed in India (51.8%) (Prajapati et al., 2017) and South Africa (43 and 53%) (Pietersen et al., 2015; Yuengling et al., 2018). The relatively low mortality rate in the aforementioned studies could be due to the hiding of death by high LTUF rate ranging from 14.81 to 37.7% in these studies (Kliiman and Altraja, 2009; Frank et al., 2019; Yunusbaeva et al., 2019) as compared to 7.7% in our cohort. In the multivariate analysis, elderly patients (>60 years old) were significantly more likely to die. The combination of various risk factors like general physical deterioration, multiple comorbidities, complex medication schedule and poor immunity make the elderly DR-TB patients more prone to death. Similar positive association between older age and unsuccessful treatment outcomes among XDR-TB (Frank et al., 2019) and MDR-TB patients have been reported by studies conducted in Pakistan (Ahmad et al., 2015; Khan et al., 2019) and elsewhere (Kurbatova et al., 2012).

In the present study, there was a statistically significant negative association between death and the patients’ baseline body weight of ≥40 kg. This implies that XDR-TB patients with a baseline body weight of <40 kg were significantly more likely to die than those with a baseline body weight of ≥40 kg. Similar to our finding, the patients’ low body weight/body mass index at baseline visit has been reported as a risk factor for poor treatment outcomes among XDR-TB (Kwon et al., 2008; Jacobson et al., 2010; Tang et al., 2013; Prajapati et al., 2017) and MDR-TB patients (Ahmad et al., 2015; Javaid et al., 2017; Khan et al., 2019) by studies conducted elsewhere. One of the reasons of poor systemic availability of oral anti-TB drugs is their poor absorption from gastrointestinal tract (GIT). Malnourishment in TB patients not only contributes to poor GIT absorption of anti-TB drugs (Byrd et al., 2002) but also increases the urinary excretion of free drugs, consequently leading to sub-therapeutic serum drug concentration (Pinheiro et al., 2006) and unsuccessful treatment outcomes.

In the current cohort, achieving SCC in the initial two months of treatment had inverse relationship with death. In pulmonary TB patients, achieving SCC is an important and early indicator of non-infectiousness of the patient and effectiveness of the treatment regimen (Pinheiro et al., 2006; Javaid et al., 2018). Due to its predictive value in determining cure, SCC is used as an early microbiological end point in phase II clinical trials of TB treatment. Similar finding regarding early SCC and successful treatment outcomes among XDR-TB patients (Prajapati et al., 2017; Frank et al., 2019; Te Riele et al., 2019) and MDR-TB patients (Kurbatova et al., 2015; Javaid et al., 2018) have been reported elsewhere. Therefore, like in the case of MDR-TB, predicting validity of time to SCC and factors associated with it among XDR-TB patients can help doctors in identifying patients at high risk of poor outcomes early in the course of XDR-TB treatment.

Those patients of the current cohort who were taking high dose isoniazid were 2.3 times more likely to die than their counterparts. However, the previously published studies among XDR-TB patients have not reported any such finding. Following the assumption that high dose isoniazid may be effective in *MTB* strains with low-level isoniazid resistance due to mutations in the inhA promotor at positions 8, 15 or 16 (Domínguez et al., 2016), WHO guidelines proposed the high-dose isoniazid in the treatment of MDR/XDR-TB patients (World Health Organization, 2016). However, there is a general consensus that high-dose isoniazid treatment cannot overcome the high-level isoniazid resistance caused by mutation in the *katG* gene at position 315 (Domínguez et al., 2016; Chesov et al., 2017). A study from Republic of Moldova which evaluated the molecular drug-resistance in 2638 MTB strains found that mutation in the katG gene at position 315 was present in 88.1% of the tested strains (Chesov et al., 2017). As the use of high dose isoniazid may be associated with higher incidence of hepatitis, peripheral neuropathy and other unforeseen adverse effects (World Health Organization, 2016), therefore, the indiscriminate use of high dose isoniazid in DR-TB in the absence of comprehensive molecular drug resistance testing should be discouraged (Chesov et al., 2017). Nevertheless, the current finding of positive association between the used of high dose isoniazid and death among XDR-TB patients should be interpreted with the major limitation that only 41 (10.1%) patients of the current cohort received high dose isoniazid. Furthermore, these patients who received high dose isoniazid might also have more severe form of TB. Therefore, a study with large number of XDR-TB patients receiving high dose isoniazid is suggested to confirm the current finding.

In the current study, the LTFU rate (7.7%) is in line with studies from China (5.9%) (Tang et al., 2013), South Africa (7.0%) (Pietersen et al., 2015) and India (9.8%) (Prajapati et al., 2017), but lower than the rates reported by studies conducted in Georgia (37.7%) (Frank et al., 2019), South Africa (16.2%) (Yunusbaeva et al., 2019) and Estonia (14.8%) (Kliiman and Altraja, 2009). In the present study, 24/31 patients were LTFU prior to SCC. As those patients who are sputum culture positive are highly infective and potential source of XDR-TB transmission, LTFU prior to SCC is a serious threat to the public health. In multivariate analysis, male gender emerged as the only risk factor for LTFU. As majority of the patients in current cohort were of economically productive age group, and in developing countries like Pakistan young men usually earn daily living by doing labor work or migration for employment. These factors along with conflicting working hours make it challenging for them to adhere with the prolonged treatment of XDR-TB. Likewise positive association between male gender of TB patient and LTFU has been reported by a study conducted elsewhere (Dooley et al., 2011).

## Conclusion

The rate of successful treatment outcomes (40.6%) in the current cohort was comparable to the global treatment success rate (39%) among XDR-TB patients, but far lower than the target (75%) set by the WHO. The variables which emerged as risk factors for death and LTFU in the current cohort i.e., patients’ age of >60 years, receiving high dose isoniazid, baseline body weight of <40 kg, failure to achieve SCC in the initial two months of treatment and male gender are easily recognizable before diagnosis or in the very beginning of XDR-TB treatment. Giving special attention and enhanced clinical management, nutritional supplementation, strategies to ensure treatment completion by and decentralizing the treatment in patients at high risk of death and LTFU may improve the treatment outcomes. Therapeutic drug monitoring in those XDR-TB patients who had baseline body weight <40 kg may help in the dose adjustment and manipulation anti-TB therapy in these patients. By evaluating the treatment outcomes of 404 XDR-TB patients from 27 PMDT units all over the country and following the WHO standard methodology, the current study can be considered as a representative of the entire XDR-TB population from Pakistan. However, the observational design, retrospective nature of data collection, convenient sampling method and inability to objectively assess the patients’ adherence with therapeutic regimen are the major limitations associated with this study. This study also lacks information about the radiological findings, body mass index, and incidence and management of adverse events in the current cohort which have previously been reported as factors associated with treatment outcomes in DR-TB patients. Furthermore, life events of patients that can influence an unsuccessful treatment outcome (i.e., lost to follow-up, death or treatment failure) were not recorded and analyzed.

## Data Availability

All data gathered or analyzed during this study are included in the article/Supplementary Material.
