# Supplementary material for: Treatment Outcomes of Extensively Drug-Resistant Tuberculosis in Pakistan: A Countrywide Retrospective Record Review
Source: Front Pharmacol. 2021 Mar 31;12:640555. doi: 10.3389/fphar.2021.640555 (PMC8044444; doi:10.3389/fphar.2021.640555)
Supplement: Supplementary file 1 [file datasheet1.docx]

**Supplementary table 1. PMDT site wise distribution of study participants**

| **S. No** | **PMDT site** | **Province** | **No. (%)** |
| --- | --- | --- | --- |
| 1 | Gulab Devi Hospital Lahore | Punjab | 39 (9.7) |
| 2 | Leprosy Hospital Rawalpindi | Punjab | 39 (9.7) |
| 3 | Nishter Hospital Multan | Punjab | 34 (8.4) |
| 4 | Jinnah Hospital Lahore | Punjab | 14 (3.4) |
| 5 | Govt. Samli Sanatorium Hospital Murree | Punjab | 28 (4.5) |
| 6 | Mayo Hospital Lahore | Punjab | 12 (3.0) |
| 7 | District Headquarter Hospital Sargodha | Punjab | 10 (2.5) |
| 8 | Sheikh Zayed Hospital Rahim Yar Khan | Punjab | 8 (2.0) |
| 9 | Bahawal Victoria Hospital Bahawalpur | Punjab | 7 (6.0) |
| 10 | District Headquarter Hospital Faisalabad | Punjab | 7 (1.7) |
| 11 | Allama Iqbal Memorial DHQ Hospital Sialkot | Punjab | 6 (1.5) |
| 12 | Military Hospital Rawalpindi | Punjab | 4 (1.0) |
| 13 | Abbas Institute of Medical Sciences Hospital Muzaffarabad | AJK | 3 (0.7) |
| 14 | Indus Hospital Karachi | Sindh | 48 (11.9) |
| 15 | Ojha Institute of Chest Disease Karachi | Sindh | 50 (12.4) |
| 16 | Institute of Chest Disease Kotri | Sindh | 17 (4.2) |
| 17 | Ghulam Muhammad Meher Civil Hospital Sukkur | Sindh | 15 (3.7) |
| 18 | District Headquarter Hospital Mirpurkhas | Sindh | 12 (3.0) |
| 19 | Jinnah Postgraduate Medical Center Karachi | Sindh | 2 (0.5) |
| 20 | District Headquarter Hospital Tharparkar | Sindh | 3 (0.7) |
| 21 | Chandka Medical College Larkana | Sindh | 1 (0.2) |
| 22 | Peoples Medical College Nawabshah | Sindh | 1 (0.2) |
| 23 | Fatima Jinnah Chest Hospital Quetta | Balochistan | 8 (6.9) |
| 24 | Lady Reading Hospital Peshawar | KPK | 28 (6.9) |
| 25 | Mufti Mehmood Memorial Teaching Hospital DI Khan | KPK | 10 (2.5) |
| 26 | Ayub Teaching Hospital Abbottabad | KPK | 6 (2.2) |
| 27 | Saidu Sharif Teaching Hospital Swat | KPK | 2 (0.5) |

AJK, Azad Jammu and Kashmir; KPK, Khyber Pukhtoonkhwa

**Supplementary table 2: Definitions of treatment outcomes**

| **Outcome** | **Definitions** |
| --- | --- |
| Cured | A smear positive patient who completed his/her treatment with no evidence of treatment failure, and had three or more consecutive negative cultures taken at least 30 days apart after the intensive phase of treatment. |
| Completed | The patient who completed his/her treatment as recommended by the guidelines with no evidence of failure but with less than 3 consecutive negative cultures taken at least 30 days apart after the intensive phase of treatment. |
| Death | When a patient died for any reason during the course of XDR-TB treatment. |
| Failure | The outcome “failure” was assigned to anyone of the following patients: (i) lack of SCC by the end of extended intensive phase (12 months) (ii) treatment discontinued because of poor clinical and radiological response or adverse event (iii) culture reverted (to positive) after an initial conversion; two consecutive cultures were taken at least 30 days apart are found to be positive during the continuation phase. |
| Lost to follow up | A patient whose treatment was interrupted for two consecutive months or more |
