# Supplementary material for: Treatment Outcomes of Extensively Drug-Resistant Tuberculosis in Pakistan: A Countrywide Retrospective Record Review
Source: Front Pharmacol. 2021 Mar 31;12:640555. doi: 10.3389/fphar.2021.640555 (PMC8044444; doi:10.3389/fphar.2021.640555)
Supplement: Supplementary file 2 [file datasheet2.docx]

**Data Collection Form**

| **A. Patient Information and socio-demographic characteristics** | | | | | | | |
| --- | --- | --- | --- | --- | --- | --- | --- |
| **Pt Code** | | |  | | | | |
| **Gender** | | | □ Female □ Male | | | | |
| **Age** (Years) | | |  | | | | |
| **Marital status** | | | □ Married □ Un-married □ Widow | | | | |
| **Nationality** | | | □ Pakistani □ Afghan | | | | |
| **Weight** (kg) | | |  | | | | |
| **Smoking status** | | | □Never □Ex smoker (quit > 30 days)  □Current and active | | | | |
| **Close contacts status** | | | □ No TB □ DR-TB □ Susceptible TB | | | | |
| **Employment status** | | | □ No □ Yes | | | | |
| **B. Registration** | | | | | | | |
| **Cat IV treatment Reg. No** | | |  | | | | |
| **Address** | | |  | | | | |
| **Date of Cat IV Reg.** | | |  | | | | |
| **C. Disease description** | | | | | | | |
| **Site** | | | □Pulmonary □Extra-pulmonary  □If Extra-pulmonary specific site__________________ | | | | |
| **Other Medical diagnosis** | | |  | | | | |
| **D. Registration group** | | | | | | | |
| □New | | | | □Relapse | | □Treatment after default | |
| □ Failure of Cat-I treatment | | | | □ Failure of Cat-II treatment | | □Transfer in | |
| □Others | | | | | | | |
| **E. Previous TB Treatment** | | | | | | | |
| **Previous Treatment center** | □ Public □ Private □ PPM | | | | | | |
| **No.** | **Start Date** | | | | **Regimens** | | **Outcome** |
|  |  | | | |  | |  |
|  |  | | | |  | |  |
|  |  | | | |  | |  |
|  |  | | | |  | |  |
| **Used 2^nd^ line drugs previously** | □Yes □No  □If Yes Specify_________________________ | | | | | | |
| **F. HIV information** | | | | | | | |
| **HIV testing done** | | □Yes □No □Unknown | | | | | |
| **Result** | | □Positive □Negative | | | | | |
| **Started on ART** | | □Yes □No □Date | | | | | |

| **G. Microbiological data** | | | | | | | | | | | | | | | | | | | |
| --- | --- | --- | --- | --- | --- | --- | --- | --- | --- | --- | --- | --- | --- | --- | --- | --- | --- | --- | --- |
| **Gene Xpert results** | | | | | | | □MTB resistant to RMP □ Only MTB detected | | | | | | | | | | | | |
| **Date of test** | | | | | | |  | | | | | | | | | | | | |
| **Month of treatment** | | **Sputum microscopy** | | | | | | | | | **Month of treatment** | | **Culture** | | | | | | |
|  |  | **Date** | | **Result** | | | | | | |  |  | **Date** | | **Result** | | | | |
| Diagnostic specimen | |  | | □ Negative □ Scanty □+1 □+2 □+3 □Others__________ | | | | | | | Diagnostic specimen | |  | | □ Negative □ Positive  □Others_________ | | | | |
| 0 | |  | | □ Negative □ Scanty □+1 □+2 □+3 □Others__________ | | | | | | | 0 | |  | | □ Negative □ Positive  □Others_________ | | | | |
| 1 | |  | | □ Negative □ Scanty □+1 □+2 □+3 □Others__________ | | | | | | | 1 | |  | | □ Negative □ Positive  □Others_________ | | | | |
| 2 | |  | | □ Negative □ Scanty □+1 □+2 □+3 □Others__________ | | | | | | | 2 | |  | | □ Negative □ Positive  □Others_________ | | | | |
| 3 | |  | | □ Negative □ Scanty □+1 □+2 □+3 □Others__________ | | | | | | | 3 | |  | | □ Negative □ Positive  □Others_________ | | | | |
| 4 | |  | | □ Negative □ Scanty □+1 □+2 □+3 □Others__________ | | | | | | | 4 | |  | | □ Negative □ Positive  □Others_________ | | | | |
| 5 | |  | | □ Negative □ Scanty □+1 □+2 □+3 □Others__________ | | | | | | | 5 | |  | | □ Negative □ Positive  □Others_________ | | | | |
| 6 | |  | | □ Negative □ Scanty □+1 □+2 □+3 □Others__________ | | | | | | | 6 | |  | | □ Negative □ Positive  □Others_________ | | | | |
| 7 | |  | | □ Negative □ Scanty □+1 □+2 □+3 □Others__________ | | | | | | | 7 | |  | | □ Negative □ Positive  □Others_________ | | | | |
| 8 | |  | | □ Negative □ Scanty □+1 □+2 □+3 □Others__________ | | | | | | | 8 | |  | | □ Negative □ Positive  □Others_________ | | | | |
| 9 | |  | | □ Negative □ Scanty □+1 □+2 □+3 □Others__________ | | | | | | | 9 | |  | | □ Negative □ Positive  □Others_________ | | | | |
| 10 | |  | | □ Negative □ Scanty □+1 □+2 □+3 □Others__________ | | | | | | | 10 | |  | | □ Negative □ Positive  □Others_________ | | | | |
| 11 | |  | | □ Negative □ Scanty □+1 □+2 □+3 □Others__________ | | | | | | | 11 | |  | | □ Negative □ Positive  □Others_________ | | | | |
| 12 | |  | | □ Negative □ Scanty □+1 □+2 □+3 □Others__________ | | | | | | | 12 | |  | | □ Negative □ Positive  □Others_________ | | | | |
| 13 | |  | | □ Negative □ Scanty □+1 □+2 □+3 □Others__________ | | | | | | | 13 | |  | | □ Negative □ Positive  □Others_________ | | | | |
| 14 | |  | | □ Negative □ Scanty □+1 □+2 □+3 □Others__________ | | | | | | | 14 | |  | | □ Negative □ Positive  □Others_________ | | | | |
| 15 | |  | | □ Negative □ Scanty □+1 □+2 □+3 □Others__________ | | | | | | | 15 | |  | | □ Negative □ Positive  □Others_________ | | | | |
| 16 | |  | | □ Negative □ Scanty □+1 □+2 □+3 □Others__________ | | | | | | | 16 | |  | | □ Negative □ Positive  □Others_________ | | | | |
| 17 | |  | | □ Negative □ Scanty □+1 □+2 □+3 □Others__________ | | | | | | | 17 | |  | | □ Negative □ Positive  □Others_________ | | | | |
| 18 | |  | | □ Negative □ Scanty □+1 □+2 □+3 □Others__________ | | | | | | | 18 | |  | | □ Negative □ Positive  □Others_________ | | | | |
| 19 | |  | | □ Negative □ Scanty □+1 □+2 □+3 □Others__________ | | | | | | | 19 | |  | | □ Negative □ Positive  □Others_________ | | | | |
| 20 | |  | | □ Negative □ Scanty □+1 □+2 □+3 □Others__________ | | | | | | | 20 | |  | | □ Negative □ Positive  □Others_________ | | | | |
| 21 | |  | | □ Negative □ Scanty □+1 □+2 □+3 □Others__________ | | | | | | | 21 | |  | | □ Negative □ Positive  □Others_________ | | | | |
| 22 | |  | | □ Negative □ Scanty □+1 □+2 □+3 □Others__________ | | | | | | | 22 | |  | | □ Negative □ Positive  □Others_________ | | | | |
| 23 | |  | | □ Negative □ Scanty □+1 □+2 □+3 □Others__________ | | | | | | | 23 | |  | | □ Negative □ Positive  □Others_________ | | | | |
| 24 | |  | | □ Negative □ Scanty □+1 □+2 □+3 □Others__________ | | | | | | | 24 | |  | | □ Negative □ Positive  □Others_________ | | | | |
| **H. DST Results** | | | | | | | | | | | | | | | | | | | |
| **Date sample collected** | **Date DST result** | | **S** | | **H** | **R** | | **E** | **Z** | **Km** | **Cm** | **Am** | | **FQ** | | **Eto** | **PAS** | **Cs** | **Other** |
|  |  | |  | |  |  | |  |  |  |  |  | |  | |  |  |  |  |
|  |  | |  | |  |  | |  |  |  |  |  | |  | |  |  |  |  |
|  |  | |  | |  |  | |  |  |  |  |  | |  | |  |  |  |  |
| **I. Drug regimens, Date of treatment start and dosage (mg), change dosage and cessation** | | | | | | | | | | | | | | | | | | | |
| **Date** | **S** | | **H** | | **R** | **E** | | **Z** | **Km** | **Cm** | **Am** | **FQ** | | **Eto** | | **PAS** | **Cs** | **Other** | **Comments** |
|  |  | |  | |  |  | |  |  |  |  |  | |  | |  |  |  |  |
|  |  | |  | |  |  | |  |  |  |  |  | |  | |  |  |  |  |
|  |  | |  | |  |  | |  |  |  |  |  | |  | |  |  |  |  |
|  |  | |  | |  |  | |  |  |  |  |  | |  | |  |  |  |  |
|  |  | |  | |  |  | |  |  |  |  |  | |  | |  |  |  |  |

| **Month** | **Weight** |
| --- | --- |
|  |  |
| **0** |  |
| **1** |  |
| **2** |  |
| **3** |  |
| **4** |  |
| **5** |  |
| **6** |  |
| **7** |  |
| **8** |  |
| **9** |  |
| **10** |  |
| **11** |  |
| **12** |  |
| **13** |  |
| **14** |  |
| **15** |  |
| **16** |  |
| **17** |  |
| **18** |  |
| **19** |  |
| **20** |  |
| **21** |  |
| **22** |  |
| **23** |  |
| **24** |  |

| **Date** | **L. Treatment outcome** |
| --- | --- |
|  | □ Cured □ Completed □ Died □ Defaulted □ Failed □ Transferred out |
